# Attention to colors induces surround suppression at category boundaries

**DOI:** 10.1038/s41598-018-37610-7

**Published:** 2019-02-05

**Authors:** Ming W. H. Fang, Mark W. Becker, Taosheng Liu

**Affiliations:** 1 Department of Psychology, Michigan State University, East Lansing, Michigan USA; 2 Neuroscience Program, Michigan State University, East Lansing, Michigan USA

## Abstract

We investigated how attention to a visual feature modulates representations of other features. The feature-similarity gain model predicts a graded modulation, whereas an alternative model asserts an inhibitory surround in feature space. Although evidence for both types of modulations can be found, a consensus has not emerged in the literature. Here, we aimed to reconcile these different views by systematically measuring how attention modulates color perception. Based on previous literature, we also predicted that color categories would impact attentional modulation. Our results showed that both surround suppression and feature-similarity gain modulate perception of colors but they operate on different similarity scales. Furthermore, the region of the suppressive surround coincided with the color category boundary, suggesting a categorical sharpening effect. We implemented a neural population coding model to explain the observed behavioral effects, which revealed a hitherto unknown connection between neural tuning shift and surround suppression.

## Introduction

When we encounter a crowded scene, we rely on visual attention to select what is most task-relevant and minimize distraction from inconsequential input. Numerous studies have demonstrated attentional selection based on locations (‘spatial attention’)^1^ and features (‘feature-based attention’)^2^. It is now well-documented that the selected location or feature is endowed with processing priority, manifested by enhanced behavioral performance and neural responses to the selected stimuli. An important open question concerns the profile of such attentional selection, i.e., how selection of a location or a feature modulates the representation of other locations and features?

In the domain of spatial attention, early studies suggested a monotonic profile of selection (e.g., the gradient model)^3^, such that attentional modulation was strongest at the attended location and decreased monotonically with the distance from the attended location. Similarly, early single-unit studies on feature-based attention (FBA), have also proposed a gradient profile in feature space, as epitomized by the feature-similarity gain (FSG) model^4,5^. According to the FSG model, attentional modulation is a monotonic function of the difference (similarity) between the attended feature and a neuron’s preferred feature, varying gradually from enhancement to suppression. Thus for both space-based and feature-based attention it seemed that there was a monotonic decrease in attentional modulation as the distance (in physical or feature space) increased from the attend stimulus.

However, more recent studies of spatial attention that sampled locations more finely have revealed a non-monotonic profile of attention comprised of “surround suppression”, such that nearby locations are more suppressed than further locations. This local suppression produces a “Mexican hat” attentional profile, which is thought to allow better distinction between closely located targets and distractors^6–11^. Notably, sampling this Mexican hat profile at too coarse a scale can produce what appears to be a monotonic function. The finding of a non-monotonic profile in spatial attention naturally led to the question of whether there is a similar pattern of surround suppression in FBA. Although many earlier studies in humans have supported the FSG model^12–17^, they either employed only two very different features, or used coarse sampling between the cued and target feature. Thus, these FBA studies are unlikely to detect a surround suppression effect in the feature space. Indeed, with finer sampling, two recent studies have reported initial evidence for a Mexican hat profile for feature-based attention to color and orientation^18,19^. However, a number of limitations in these studies could potentially weaken their conclusions (for details see General Discussion).

**Figure 1:**
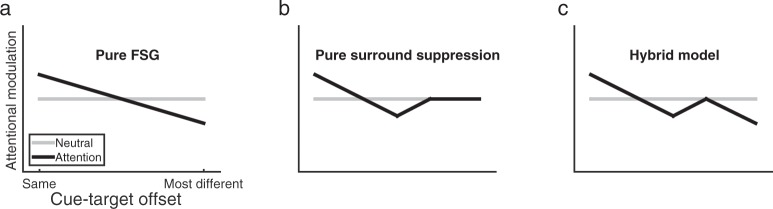
Predictions of attentional profile. The horizontal axis is cue-target offset, with the left most point (“same”) representing the attended feature and the right most point representing the most dissimilar feature (“most different”). Solid black line represents how attentional effect varies as a function of cue-target offset and gray dashed line depicts the baseline performance. (**a**) Prediction from a pure feature-similarity gain (FSG) model; (**b**) Prediction from a pure surround suppression model; and (**c**) Prediction of a hybrid model of both FSG and surround suppression at different scales. Note that the FSG induces a suppression effect at a relative larger scale while surround suppression induces a suppression effect only in the vicinity of the attended feature.

Here, we set out to determine if there is a genuine surround suppression in feature-based attention; and if so, how such a non-monotonic profile can be reconciled with a monotonic profile as stipulated by the FSG model. Based on the literature, there are three possible outcomes (Fig. 1). A pure FSG model would predict that attentional modulation monotonically decreases with cue-target dissimilarity (Fig. 1a). Alternatively, a pure surround suppression model would predict a Mexican hat profile with a complete rebound once the target feature is outside the suppressive zone (Fig. 1b). Lastly, a third possibility is a hybrid model of surround suppression at nearby features and feature-similarity gain at further features. This would predict a Mexican hat profile at the vicinity of the attended feature and a further decrease outside the suppressive zone (Fig. 1c).

We tested these theoretical predictions for color-based attention. Color is one of the most extensively studied features in attention research, perhaps because color is a particularly effective cue to guide attention^20^. Thus, it is important to know whether color-based attention elicits a surround suppression profile. Furthermore, color perception is strongly categorical such that continuous variation in input stimulus (e.g., wavelength) is mapped onto discrete hue categories (e.g., red, green, blue, etc.). This categorical nature of color perception is known to play a role in attention. For example, visual search for color targets is inefficient when target and distractors are linearly non-separable in color space^21,22^. However, such inefficiency is much reduced when targets and distractors are from different categories than if they come from the same category, even when the perceptual similarity between targets and distractors are equated^23^ (and an analogous effect in orientation)^24^. The fact that perceptual categories influence search efficiency suggests that categorical structures can modulate attention to colors. We thus conjectured that color categories could also impact the attentional profile.

**Figure 2:**
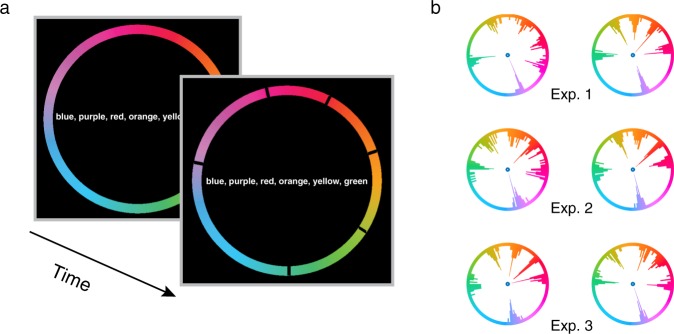
Trial sequence and example participants’ responses in the color boundary pretest. (**a**) Schematic for the trial sequence. (**b**) Examples of individual participants’ frequency distributions of clicks on the wheel. Two participants’ data for each of the three experiments were shown.

**Figure 3:**
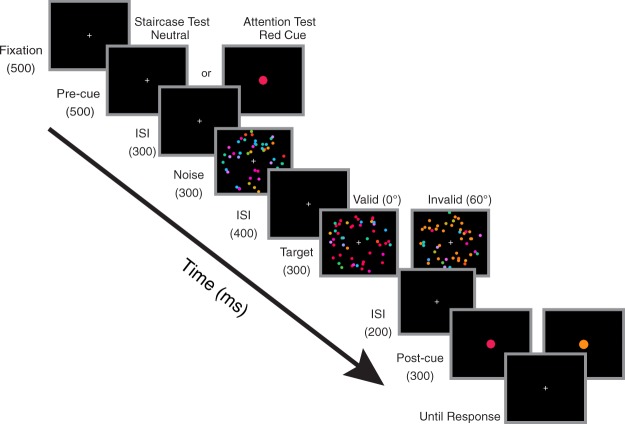
Trial sequence for the staircase and attention test in Experiment 1. The color dots are enlarged compared to the actual stimulus for illustration purpose.

In the current study, we first determined individual participant’s color categories in a pretest (Fig. 2). Participants then performed a 2-interval forced choice (2-IFC) detection task of a coherent color signal (Fig. 3). We manipulated FBA by cuing participants to attend to a particular color (attention condition) or not to attend to any particular color (neutral condition). We systematically varied the similarity between the cue and the target with a fine sampling method in the attention condition and compared detection performance to the neutral condition, to measure the attentional profile. This measurement was conducted for three different color categories to examine the impact of categorical structure on attentional profile. To foreshadow the results, we found a non-monotonic surround suppression effect for color values close to the attended one, and a feature-similarity gain effect for more dissimilar colors, consistent with the hybrid model above (Fig. 1c). Furthermore, the width of the suppressive surround coincided with the category boundaries of colors, which suggests a category-level sharpening effect to the attended color. Finally, we implemented a simple neural coding model to examine the underlying neural mechanisms of the observed attentional profile.

## Results

### Experiment 1

In this experiment, the attention cue was a red color at the center of the participant’s red category. The target was most frequently the cued color but also appeared in eight colors varying in 15° increments from ±15° to ±60° from the cued color. We compared detection performance under color cueing with the neutral condition to assess the modulation profile of color-based attention.

#### Category boundaries

**Figure 4:**
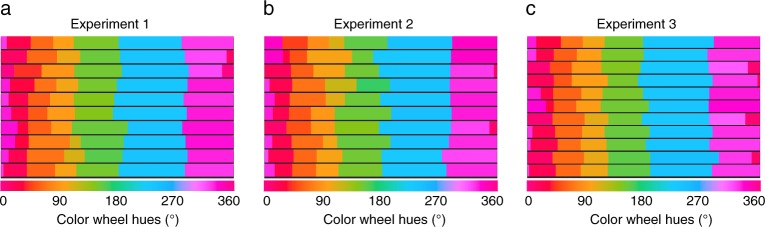
Individual category boundaries for all six color categories. Each row represents one participant’s boundaries. The labeling colors are chosen from the middle points of categories. The width of each color corresponds to the category width anchored by a participant on the color wheel. (**a**–**c**) Represent category boundaries for Experiment 1 (n = 10), 2 (n = 10), & 3 (n = 11) respectively.

Figure 4a summarizes individual participants’ boundary profile for Experiment 1. The width of color strips represents the category width on the color wheel. There was a general consistency of the locations of the color boundaries across participants, with the degree of consistency varying for different boundaries. The variability (i.e., standard deviation) across the six boundaries ranged from 3.62° (for blue-purple boundary) to 12.59° (for orange-yellow boundary). Here we used a red cue that was the individual participant’s center color in the red category. The red category was relatively narrow, with a mean span of 34.9 degrees (SD = 8.6°). Thus, the target colors at ±15° offset from the cue would be the closest to the boundaries of red category.

#### Baselines and Cueing Effect

**Figure 5:**
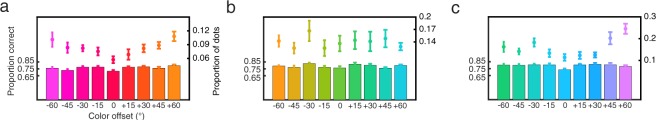
Threshold and baselines performances. (**a**) For the red color set (Exp. 1, n = 10), the bar graph represents subjects’ average baseline performances under neutral cueing condition. The dots above the bar graph represents average thresholds obtained from the staircase test. (**b**,**c**) Shows the average baseline performances and average thresholds for green (Exp. 2, n = 10) and blue (Exp. 3, n = 11) color set respectively. Error bars represent standard error of the mean.

Figure 5a depicts the average thresholds obtained from the staircase pretest and average performance across test colors in the neutral cue blocks (baseline) in the attention test sessions. The thresholding task worked fairly well to equate performance across test colors in the neutral blocks; the average baseline accuracies for all test colors were within the ±5% of the desired 75% accuracy level. We then calculated a cueing effect for each test color by subtracting the baseline performance for that color from its performance in the condition with a red pre-cue.

**Figure 6:**
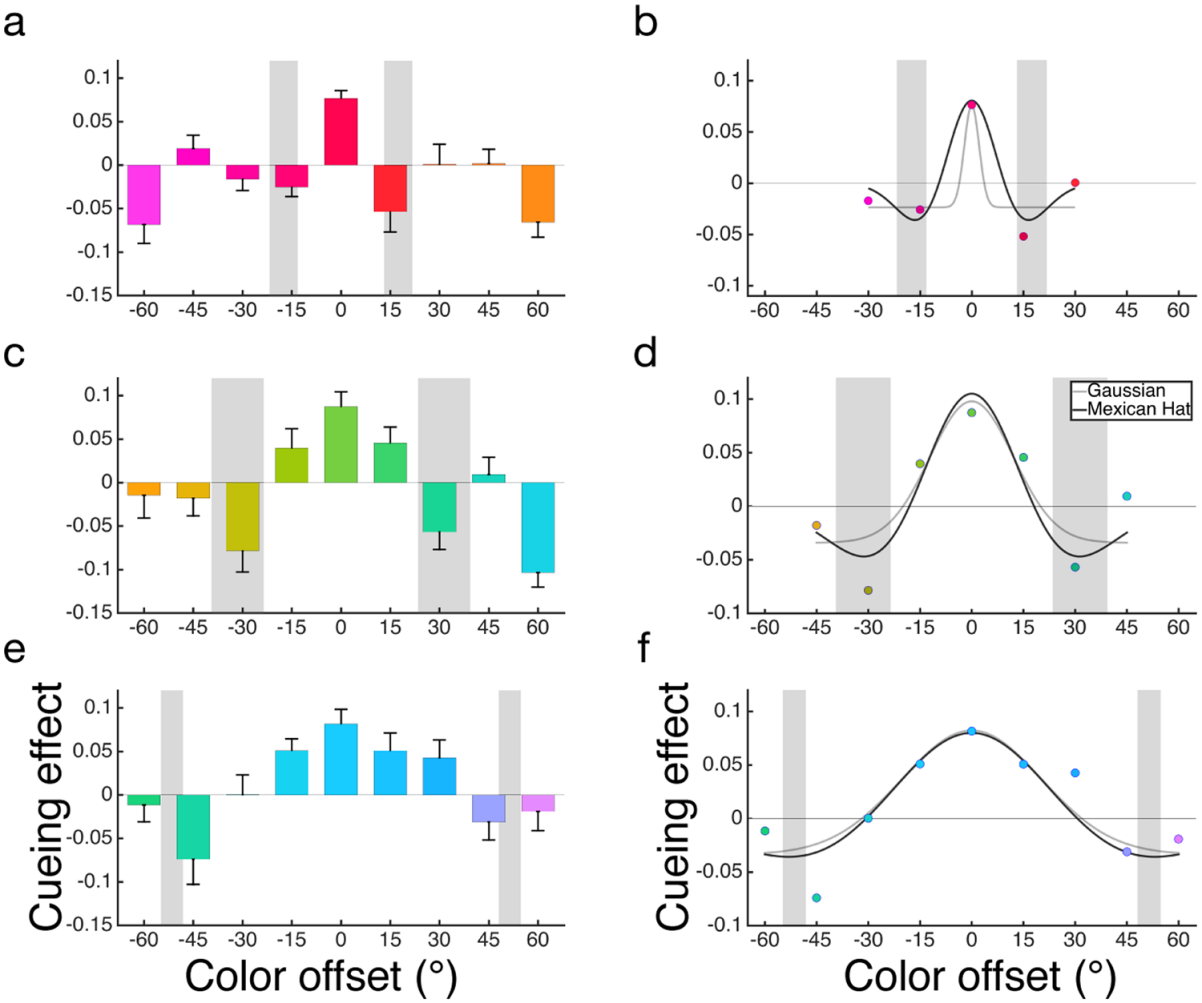
Results for Experiment 1 (n = 10), 2 (n = 10) & 3 (n = 11). (**a**,**c**,**e**) Average cueing effect for all test colors measured as performance difference between color cue and neutral condition for Experiment 1, 2, & 3 respectively. (**b**,**d**,**f**). Best fitting non-monotonic Mexican hat function and monotonic Gaussian function to the observed data. The shaded areas in all six panels represent average category boundaries ± 1 SD. Error bars represent standard error of the mean.

As illustrated in Fig. 6a, the overall pattern shows an enhancement when the red cue and test color were the same (0° offset, “valid condition”), and suppressions when they were most different (±60°, “invalid conditions”). However, the cueing effects do not follow a monotonic function of offsets between 0° and ±60°. Importantly, suppressions also emerged at the immediate neighbors (±15°) of the red cue, but not at further colors (±30° and ±45°). To better characterize the shape of the cueing effects, we performed two further analyses.

**Figure 7:**
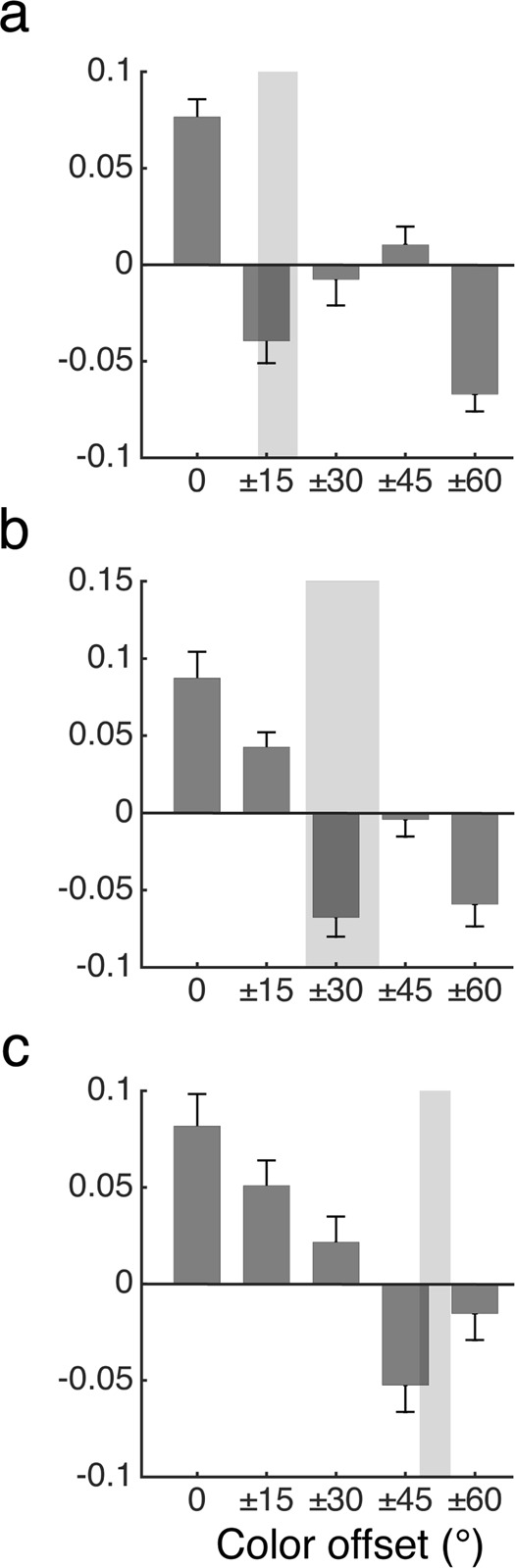
Combined cueing effects. For each of the three color sets (i.e., red, green, and blue), we averaged the cueing effect across pairs of positive and negative offsets. Panels (a–c) show results from Experiments 1 (n = 10), 2 (n = 10), and 3 (n = 11), respectively. The shaded areas represent mean boundaries ± 1 SD. As the boundaries width increased, the suppressive surround also increased and overlapped with the boundary areas. Error bars represent standard error of the mean.

*Combined cueing effect*. To further summarize the cueing effect, we averaged the participant-level cueing effect for each pair of +/− cue-target offset (Fig. 7a), and then conducted one-sample t-tests against zero to assess attentional enhancement and suppression. We used Bonferroni corrected alpha (at 0.01 level) for statistical inferences due to multiple comparisons. There was a significant enhancement at 0° cue-target offset, t(9) = 8.38, *p* = 1.5 × 10^−5^, Cohen’s d = 2.65, CI = (0.056,0.097) a significant suppression at 15° offset, t(9) = −3.38, *p* = 0.0081, d = 1.07, CI = (−0.065, −0.013). However, the cueing effect was non-significant at both 30° (t(9) = 0.62, *p* = 0.55) and 45° (t(9) = 0.91, *p* = 0.39) offsets. At the maximum 60° offset, there again was a significant suppression, t(9) = −7.60, *p* = 3.4 × 10^−5^, d = 2.4, CI = (−0.088, −0.047).

*Model Comparison:* As a complementary analysis to the significance tests above, we also fitted both the monotonic (i.e., Gaussian) model and the non-monotonic (i.e., Mexican-hat) model to the average cueing effects (See Fig. 6b). For this analysis, the largest offsets that we included were those showing an immediate rebound in cueing effect (±30°) following suppressions. Our general approach was to exclude data points outside of the immediate rebound as these points were not part of the surround suppression (see Fig. 1c). In addition, our previous work suggests that feature-similarity gain suppression can occur at 60° offset^16^. Thus, for the model fitting, we excluded ±45° and ±60°. Within the range between −30° to +30° offsets, the Mexican hat model (R^2^ = 0.94) is favored by a Bayes factor of 21.73 over the Gaussian model (R^2^ = 0.85), which constitutes strong evidence^25^ for the Mexican hat model. Similarly, the AIC evidence also supported the Mexican hat model as 26.03 times more likely than the Gaussian model. We also conducted similar model comparisons for individual participants’ data and found that the Mexican hat model was favored in 7 out of 10 participants based on both AIC and BIC model evidence.

### Discussion

In this experiment, we measured individual participant’s category boundaries and selected test colors (i.e., cue and target colors) based on the center of each participant’s red category. By manipulating the cue-target offset at a fine scale (every 15 ° on the color wheel), we found a significant suppression effect at the immediate neighbors (±15°) of the cued color, but not at its further neighbors (±30° and ±45°). These results support a surround suppression model of attentional modulation when attending to a color. However, at a larger scale there was also a significant suppression at ±60° offset, which is consistent with a feature-similarity gain modulation when colors become more dissimilar^16^. Thus, the overall pattern of results shows both effects: surround suppression and feature-similarity gain (Fig. 1c). Interestingly, we found that the suppressive surround coincided with the category boundaries (see Fig. 6b, shaded area represents the average boundaries ±1 SD), suggesting a potential link between surround suppression and categorical color perception.

### Experiment 2

In this experiment, we further examined the attentional profile for colors and the relationship between color categories and surround suppression, by testing a different color category. The boundary pretest results in Experiment 1 showed that the green category was roughly twice as wide as the red category, and thus we chose to test the green category to investigate whether the suppressive zone remained at a constant extent or scaled with the width of the category. The methods were identical to those in Experiment 1, except the colors were selected around individual participant’s green category.

#### Category boundaries

We found generally consistent boundary placements across participants (Fig. 4b). The variability (i.e., standard deviation) of individual participants’ boundaries ranged from 4.74°(blue-purple) to 11.59°(orange-yellow). The center point of each individual participant’s green category was chosen as the color cue. The green category had an average width of 62.9° (SD = 15.95°), with its boundaries closest to target colors at ±30° offset.

#### Baselines and Cueing Effect

Figure 5b depicts the average thresholds from the pretest and baseline performance (in neutral cue blocks) in the attention test sessions. The average baseline accuracies for all test colors were within ±7.5% range of the desired 75% accuracy level.

Figure 6c shows the overall cueing effect for the green category. There was both an enhancement at 0° and a suppression at +60°, with a less pronounced suppression at −60°. Moreover, the cuing effect was non-monotonic; suppressions emerged at ±30° but rebounded at ±45°. To verify the non-monotonic pattern, we conducted two further analyses.

*Combined cueing effect:* We averaged the cueing effect along the positive and negative offsets to further summarize the cueing effect (Fig. 7b). We found a significant enhancement at cue-target offsets of 0°, t(9) = 5.18, *p* = 5.8 × 10^−4^, Cohen’s d = 1.64, CI = (0.05, 0.13), and 15°, t(9) = 4.45, *p* = 0.0016, d = 1.41, CI = (0.021,0.064). For the 30° offset, there was a significant suppression, t(9) = −5.41, *p* = 4.3 × 10^−4^, d = 1.71, CI = (−0.096, −0.04), but not at 45° offset, t(9) = −0.38, *p* = 0.71. At the larger offset of 60°, the suppression again reached significance, t(9) = −4.08, *p* = 0.0028, d = 1.3, CI = (−0.092, −0.026).

*Model comparison:* Following the same model fitting procedure as in Experiment 1, we excluded cue-target offset at ±60° because they were outside of the immediate rebound at ±45°. As shown in Fig. 6d, the Mexican hat model (R^2^ = 0.84) was favored with a Bayes factor of 20.48 over the Gaussian model (R^2^ = 0.71), again constituting strong evidence for the Mexican hat model. Similarly, the AIC model evidence favored the Mexican hat model 21.22 times over the Gaussian model. For individual participants, the Mexican hat model was favored in 9 out of 10 participants based on both AIC and BIC model evidence. An overlap was also observed between the suppressive surround and the boundary areas of the green category (shaded regions).

### Discussion

Results in Experiment 2 replicated the findings in Experiment 1 and provided further evidence that attention to a color elicits suppression at neighboring colors with a further rebound, i.e., the surround suppression effect. Meanwhile, at a larger scale (e.g., at 60° offsets), our current findings are again consistent with feature similarity gain modulation^16^ (Fig. 1c). Furthermore, in the current experiment, we intentionally selected a color category (i.e., green) that was wider than the red category tested in Experiment 1. As the category width increased, we also found an increase in the width of the suppressive surround; in both experiments there was an overlap between the area of surround suppression and category boundaries. Thus, the suppressive zone does not have a fixed width but tracked the color category’s boundary, suggesting at a relationship between suppression mechanism and the perception of color categories.

### **Experiment 3**

In this experiment, we aimed to further verify the potential link between surround suppression and color category boundaries. We tested a third color set around the blue category that spanned the largest range compared to the red and green categories. If attention suppresses boundary colors, we should find a further increase in the suppressive surround width for the blue color set compared to previous color sets.

#### Category boundaries

Like the two previous experiments, there was a general consistency among participants and also variability among individuals’ color boundaries (Fig. 4c). The smallest boundary variability was found in blue-purple (SD = 5.13°), while the largest was found in purple-red (SD = 14.02°). The cue color was selected as the center point of each individual participant’s blue category. The blue category had the largest average width, which spanned 102.9° (SD = 6.73°) on the color wheel. Thus, the target colors at ±45° would be located closest to the category boundaries.

#### Baselines and Cueing Effect

Figure 5c depicts the average thresholds from the pretest and baseline performance with the neutral cue in the attention test sessions. The baseline accuracies for all test colors were within ±6.2% range of the desired 75% accuracy level.

The overall cueing effect is shown in Fig. 6e. Similar to the previous two experiments, there was a significant enhancement at 0° offset and an overall non-monotonic pattern such that colors at ±45° had the lowest accuracy, and a further rebound was observed at larger offset (±60°). However, this pattern was more pronounced around the green-blue boundary than the blue-purple boundary. To verify the non-monotonic pattern, we conducted two further analyses.

*Combined cueing effect*: Following the same procedure in the Experiment 1 & 2, we averaged the cueing effect along the positive and negative color offsets (Fig. 7c), and found a significant enhancement for both 0° and 15° offsets (t(10) = 4.93, p = 6 × 10^−4^, Cohen’s d = 1.49, CI = (0.045, 0.12), and t(10) = 3.89, p = 0.003, d = 1.17, CI = (0.02, 0.08) respectively). Furthermore, only colors at 45°, t(10) = −3.77, p = 0.0036, d = 1.14, CI = (−0.084, −0.022), but not 60° (t(10) = −1.11, *p* = 0.29), were significantly suppressed.

*Model comparison*: Given the suppressive surround was observed at ±45°, we fitted both the non-monotonic and monotonic function to all data points (i.e., including ±60° offsets) to include the rebound part of the profile (Fig. 6f). The model comparison result provided positive support^25^ for the Mexican hat model (R^2^ = 0.79) over the Gaussian model (R^2^ = 0.76) with a Bayes factor of 4.85 based on BIC evidence, and with a likelihood ratio of 4.41 with AIC evidence. For individual participants, the Mexican hat model was favored by 9 and 8 (out of 11) participants based on BIC and AIC evidence, respectively. Importantly, the suppressive surround once again scaled with color category width and emerged near the boundaries of blue category.

### Discussion

Once again, we found positive support for the Mexican hat model in a new set of colors around the blue category. Somewhat unexpectedly, the surround suppression effect in the current experiment is asymmetric: we found a stronger suppression effect around green-blue category than the blue-purple boundary. It is not clear why this occurred, but we note that of all the color categories we tested here, the blue/purple category might be the most qualitatively distinct, which may have impacted the exact profile of surround suppression. Overall, our model comparison and the analysis of the combined cueing effect further verified that such non-monotonic pattern reflected genuine suppression effects. In addition, as the test category’s width increased, we again found a close mapping between the suppressive surround and category width. Taken as a whole, the surround suppression effect and its coupling to the category boundary are thus robust findings that are consistent across all three experiments.

Note we did not find a strong feature-similarity effect in this experiment. This is likely due to the limited range of the test colors, as the most dissimilar colors were close to the category boundaries and thus still on the rebound part of the suppressive surround. A feature similarity effect has been observed for larger color offsets in our previous study^16^.

## General Discussion

We systematically manipulated feature similarity for three sets of colors to study the profile of FBA and to examine its relationship with categorical color perception. Our results showed that the attentional cueing effect was a non-monotonic function of cue-target similarity, giving rise to a surround suppression profile of FBA. Importantly, we also found that the suppressive surround consistently tracked the location of category boundaries. These findings suggest that FBA may enhance color selectivity at the category level by suppressing similar but different colors near the boundaries. When colors were most dissimilar (60°), our results from the first two experiments also showed further suppression, which is consistent with the feature-similarity gain model^4,5^. Taken together, we conclude that both surround suppression and feature-similarity gain jointly modulate FBA’s profile, but at different similarity scales (Fig. 1c).

Two earlier studies have also reported evidence for a surround suppression effect, one for color and one for orientation^18,19^. However, we believe our methods improve on those designs, making our replication of those findings particularly compelling. First, the methods in Stormer & Alvarez’s study seems overly complex in that participants were asked to attend simultaneously to two colors with varied similarity^19^; thus, the effect may have been due to other factors associated with the need to hold and attend to two colors simultaneously. Second, participants in Tombu & Tsotsos’ study were asked to judge whether oriented stripes were straight or jagged^18^, which in principle does not require orientation information. Furthermore, their effect was only observed for the jagged stripes for unknown reasons. Third, and critically, neither study measured a neutral baseline to evaluate whether there is a true suppression or simply less enhancement. Lack of a proper baseline could also lead to inaccurate characterization of the shape of attentional profile. For example, Stormer & Alvarez (2014) showed an almost complete rebound at the largest cue-target distance^19^ (similar to Fig. 1b), whereas we never observed such a complete rebound.

Building on these previous studies, we aimed to better investigate the attentional profile for color-based attention. To reduce complexity, we directly manipulated the signal strength through color coherence and cued participants to attend to a single color. To quantify attentional effect, we established baseline performance by equating perceptual differences across the test colors. We also used a post-cue to reduce response uncertainty, so that performance should reflect FBA’s modulation on perception instead of post-perceptual processes^26,27^. While our finding of surround suppression is generally consistent with initial studies reporting this effect, there are also some important differences. First and foremost, our results show a hybrid modulation of both surround suppression and feature-similarity gain on different scales, which reconciles previous literature showing the two qualitatively different attentional profiles in separate studies^16–19^. Second, we discovered that the suppressive zone coincides with the color category boundary, which has not been considered in previous studies. Stormer & Alvarez^19^ selected random colors and reported a surround suppression effect at 30°, whereas our suppressive zone varied according to the cued color category. We believe that their finding could be due to averaging of variable suppressive surround widths across different categories.

In general, attentional suppression serves to enhance signal-to-noise ratio by excluding irrelevant features. The FSG-induced suppression can exclude very dissimilar distracters, whereas surround suppression can exclude distracters that are similar and thus easily confusable with the target feature. In the case of color, our observation of the overlap between suppressive zone and category boundaries further suggests that the latter effect is equivalent to a sharpening effect at the category level. In other words, color categories might be the natural substrate to instantiate surround suppression. We thus prose that both types of suppression would facilitate efficient selection of a target feature, depending on its similarity to distracter features. The category-based surround suppression effect also supports our initial conjecture based on the finding that categorical difference facilitates search for linearly non-separable colors^23^. It is conceivable that this effect in visual search can be achieved by a sharpening effect at the category level.

Finally, we would like to point out that stimulus competition is likely necessary in order for the suppression effects to emerge. Indeed, a number of early studies found that FSG effects are only present when there is stimulus competition^14,15,28^. Likewise, both our study and previous ones on surround suppression^18,19^ mixed task-irrelevant features with target features to introduce stimulus competition. This observation is generally consistent with the biased-competition framework of attention^29^, which proposes that a key function of attentional selection is to resolve stimulus competition. Our results further extend and refine the framework by specifying the profile of such biased influence in feature-based selection.

What are the possible underlying mechanisms that generate our observed attentional profile and in particular, the surround suppression effect? While feature-similarity gain modulation has been directly observed in sensory neurons’ responses, a neuronal correlate of surround suppression has not been reported, and indeed, is apparently absent in single-unit’s response^4,5^. In the absence of direct physiological data, computational models can provide useful insights on the underlying mechanism.

One possibility is the selective tuning (ST) model, a multi-layered neural network that emulates the hierarchical visual pathway^10,11^. The original ST model was proposed to accommodate visual processing in the spatial domain (e.g., crowding, spatial resolution) and follows a hierarchical structure with increasing receptive field sizes at higher levels of the network. Critically, the model assumes a top-down feedback modulation which progresses backward along the visual hierarchy and inhibits units less tuned to the attended location in earlier layers. This top-down influence can produce spatial surround suppression in early units, and is able to account for findings in the spatial domain^6–9^. However, such an account might be less plausible in the feature domain given the lack of parallel architecture in spatial and feature processing. In the case of color, high-level neurons have been reported to exhibit quite narrow tuning to hues^30,31^. Thus, if the ST model relies on the general architecture of responses pooling across the hierarchical levels, it might not easily accommodate feature-based surround suppression. Furthermore, it is also not obvious how the ST model can account for our observed association between category boundary and surround suppression.

**Figure 8:**
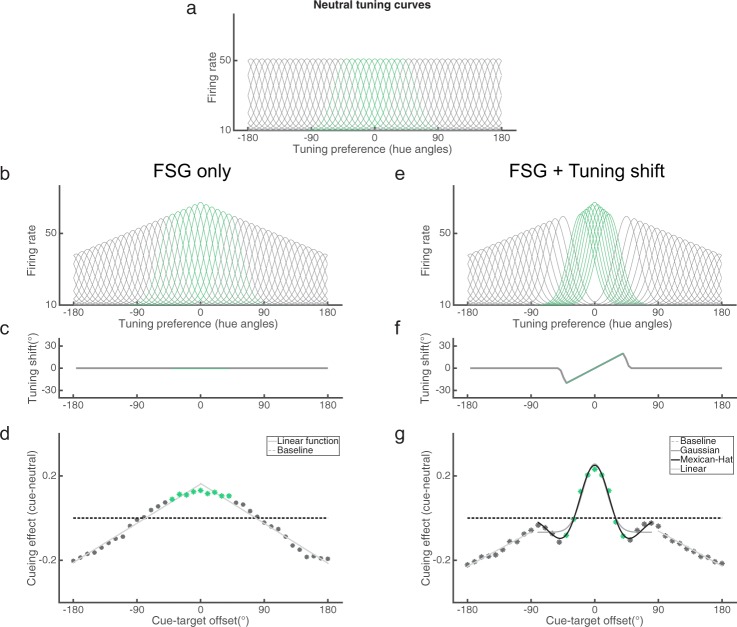
A neural model for surround suppression. (**a**) Tuning curves for a subset of simulated neurons. Green curves represent neurons within a color category (±40°). (**b**) Tuning curves under a pure FSG modulation. (**c**) Tuning shift function under the pure FSG condition, which is always zero. (**d**) Behavioral cueing effect due to FSG (black dots) monotonically decreases and is fitted with a linear function (gray lines). (**e**) Tuning curves under a hybrid modulation of both FSG and tuning shift. (**f**) Tuning shift function under the hybrid model. (**g**) Behavioral cueing effect in the hybrid model exhibits a non-monotonic profile that is better fitted by a Mexican-hat function (black solid line) than a Gaussian function (dark gray solid line). Once outside the suppressive zone, the cueing effect further decreases (light gray solid line).

Here we consider an alternative modeling approach in the framework of population neural coding and read-out with Bayesian estimation^32–35^. We aimed to build a simple model under known physiological constraints (for model details, see the Supplementary Materials). The model contains a bank of color-tuned neurons spanning the space on the color wheel (Fig. 8a). We simulated individual trials of our 2-IFC task by generating two random dot color stimuli on each trial and obtaining population neural responses for each stimulus. Similar to the 2-IFC task in human participants, the model based its decision by choosing the interval that contained a stronger color signal. We first simulated the model under a neutral condition without any attentional modulation. To check the model’s performance, we varied the coherence of the color signal and observed a better model performance with higher color coherence, similar to human participants (see Supplementary Fig. S1). For the main simulations, we used a fixed color coherence value (0.1) as it produces an intermediate performance level, to emulate the thresholding procedure in human participants.

We then simulated the model under the attention condition and evaluated how different neural-level modulations impact behavioral performance in the 2-IFC task. In these simulations, we assumed the attended color was at 0°. We first considered a feature-similarity gain modulation, where the neuronal gain is a monotonic function of the similarity between the cued color and a neuron’s preferred color (Fig. 8b). When we systematically varied the input stimuli to have different color offsets from 0°, we obtained a monotonic performance profile (Fig. 8d). This result indicates that a pure FSG mechanism cannot produce surround suppression and hence implies the existence of additional neural modulations. Here we consider a physiologically plausible mechanism – shift of neuronal tuning toward the attended feature. Previous studies have reported neuronal tuning shifts toward the attended feature during FBA^36,37^. Although tuning shift is generally interpreted as a mechanism to enhance the representation of task-relevant feature (i.e., matched filter), its connection to surround suppression has not been considered. We implemented tuning shift in our model along with FSG modulation (Fig. 8e). We assumed that the tuning shift is concentrated around the category boundary, such that within the category the shift was a constant percentage of the distance between the cued color and the neuron’s preferred color, and it was flanked by a decline outside the cue’s category (Fig. 8f). Under this scenario, the model performance exhibited a surround suppression effect at the category boundaries as well as further suppression for more dissimilar colors (Fig. 8g). We provide more in-depth explanations of the model’s behavior based on modulations of population neural responses in the Supplementary Material (see Supplementary Figs S2 and S3). Importantly, when the category boundary width changes, the location of suppressive surround shifts, such that it follows the category boundary, similar to our psychophysical findings. Thus, this simple model assumption of the extent of tuning shift can produce our observed association between surround suppression and color category.

We also explored other possible neuronal modulations to generate surround suppression. For example, we were able to produce the surround suppression effect by assuming attention sharpened the tuning curves of individual neurons within the category (but not outside the category). However, given there is no physiological evidence of sharpening of neuronal tuning curves, we consider it as a less likely candidate. Thus, based on our current model, we postulate that neuronal tuning shift is a possible mechanism for surround suppression. Critically, the modeling exercise showed that one does not need to postulate a Mexican hat modulation on individual neuron’s tuning curve to obtain a behavioral surround suppression effect. Our model’s performance is due to a simple shift in individual neuron’s tuning curves, which, at the population level, is equivalent to a Mexican hat modulation. We thus suggest that the category structure determines the pattern of tuning shift, which naturally produces surround suppression at the boundary.

Lastly, a natural question that arises from this work is whether language plays a role in our results. Specifically, because humans have verbal labels for different colors, is it possible that the category-based surround suppression effect is due to linguistic factors? Although this is a possibility, we think this is unlikely for the following reasons. First, it is still controversial whether language plays a role in the perception of color categories, with evidence both for^38^, and against the role of language^39,40^. Second, there are features in our data that do not easily fit with a linguistic interpretation. For example, we found a graded effect of attention within a category (Fig. 5b,c, smaller cueing effect for uncued green and blue colors). If linguistic concepts played a primary role, we might expect a fairly sharp category boundary without much variation within a category. Further research is necessary to examine the role of language in shaping the profile of color-based attention.

## Conclusion

We found both a surround suppression modulation and a feature-similarity gain modulation of FBA. On a large scale, attention to a color sharpens perceptual feature selectivity by suppressing most dissimilar features. On a finer scale, attention can also further enhance our perception by suppressing similar but different colors near category boundaries – a sharpening effect at the category level. We further demonstrated with model simulations that this association between suppressive surround and color category can be explained by shifting in neuronal tuning that obeys the categorical structure. Although our model is likely over-simplified and incomplete, the linkage between shifts of neuronal tuning and surround suppression is highly intriguing and could be interesting avenue for future research.

## Methods

### Participants

All participants (Exp. 1, N = 10; Exp. 2, N = 10; Exp. 3, N = 11) gave informed consent and were compensated at the rate of $10 per hour. All participants had normal or corrected-to-normal visual acuity and reported normal color vision, which was verified with the Dvorine Pseudo-Isochromatic Plates^41^. Experimental protocols were approved by the Institutional Review Board at Michigan State University. All experiments were performed in accordance with approved guidelines and regulations.

### Apparatus

The stimuli were generated using Matlab (MathWorks, Natick, MA) and MGL (http://gru.stanford.edu/mgl) and presented on a 21-in CRT monitor (1024 × 768 pixels, 100-HZ refresh rate) at a viewing distance of 69 cm. The monitor was calibrated using an I1 Pro spectrophotometer (Xrite, Grand Rapids, MI). We used the calibration procedure to linearize the luminance (gamma correction) and convert color coordinates in CIE L*a*b* color space to monitor RGB values with a white point measured as the display’s white background^42^.

### Stimuli

During the color boundary pretest, an annulus color wheel (inner radius = 9°, outer radius = 10°) was present at the center of the screen on a black background. The wheel consisted of 180 evenly-spaced hues from a circle in CIE L*a*b color space (radius = 91, luminance = 75, a = 23, b = 25). Each hue on the wheel spanned 2°. Names of color categories were displayed at the display center throughout the test. Category boundaries were labeled by black line markers (10-pixel wide) upon participant’s response (see below).

The stimuli in the main task were two static arrays of colored dots as an analog to the classic random dot motion cinematogram^43^. Each array had a total of 240 dots (radius = 0.1°), which were randomly positioned within a circular region (inner radius = 1°, outer radius = 5 °) centered on the screen. In the *noise array*, all dots had random and different colors sampled from the wheel. In the *target array*, a proportion of dots were drawn in the same color, whereas the rest were assigned colors randomly selected from the color wheel. The proportion of the same colored dots is referred to as color coherence, and these dots constitute the signal for the detection task.

### Task and procedure

Each participant performed three tasks over multiple days. They first performed a color boundary pretest, which guided stimulus color selection (see below). They then performed a baseline color coherence pretest to determine the color coherence threshold for each color used in the main task. After successfully completing the two pretests, participants performed the main attention test, which consisted of interleaved neutral pre-cue (i.e., baseline) and color pre-cue blocks to test FBA’s modulation profile. The three tasks are described in more detail below.

### Color boundary pretest

We measured category boundaries for each individual participant. Figure 2a shows an example trial sequence of the pretest. The color wheel and the names of six basic color categories (i.e., blue, purple, red, orange, yellow, and green) were always present on the screen (to avoid memory-related effects). Participants were instructed to locate the boundaries separating each of the six colors using mouse clicks. Left mouse clicks placed markers at the current cursor position and right clicks cancelled the previously placed boundary. Participants pressed the space bar after having located all six boundaries on the color wheel. Between trials, the color wheel randomly rotated clockwise or counter-clockwise (up to ±12 degrees) to exclude spatial location as cues for setting the boundaries.

Participants completed 25 trials of the pre-test (6 clicks per trial). They were told to be as accurate as possible and were given unlimited time to respond. On average, participants spent 20 minutes on this pretest. Based on each participant’s boundary data we defined 9 test colors. The category center (0° offset) was used as the color for attentional cueing (Exp 1: Red, Exp 2: Green; Exp 3: Blue), whereas 8 other colors (±15°, ±30°, ±45° and ±60° offset from the category center) were used as possible target colors (see *Analysis* below for details on how the colors were selected).

### Baseline coherence pretest

In a separate session, we measured color coherence thresholds for each of the 9 test colors for each participant, using a QUEST staircase method targeting 75% accuracy^44^. As shown in Fig. 3, each trial began with the onset of a fixation cross at the center for 300 ms, followed by a neutral precue (i.e., fixation cross) for 500 ms. After a 300 ms interstimulus interval (ISI), participants were presented the first dot array (300 ms), followed by a 400 ms ISI. Then, the second dot array appeared for 300 ms before another 200 ms ISI, followed by the post-cue (300 ms). After the post-cue disappeared, participants reported whether the first or second interval contained the target (the array with a coherent color) by pressing one of the two keys on the keyboard, which was followed by an inter-trial interval (ITI) of a blank screen for 700 ms. Each trial was randomly assigned to one of the nine staircases, such that all staircases were randomly interleaved to equate practice effect on threshold estimation.

We informed participants that the post-cue indicated the target color, in order to eliminate decision uncertainty^26,27^. All participants were given unlimited time for response. A feedback tone was given on incorrect response. Thresholds of the nine tests colors were estimated through nine staircases (72 trials per staircase), which consisted of 6 blocks of 108 trials.

### Attention (Main task)

We measured the effect of FBA on color detection with a color cue. Participants were tested with the same 2-IFC task as in the staircase test under neutral cue (i.e., fixation cross) blocks or color cue blocks. Coherence for each test color was determined by the baseline coherence test (see above). Presenting the coherence at these individually determined threshold coherence levels should have made performance relatively equal across all test colors in the neutral cue conditions. For neutral cue blocks, target colors were randomly selected from nine possible colors on each trial. For color cue blocks, a colored dot (radius = 0.2 dva) with a hue at the middle point of individual participant’s test category was presented at the screen center to orient FBA. The target color matched the cue in half of the trials (“valid condition”) so that the cue was predictive, and in the other half trials, the target color was randomly assigned to be one of the other eight test colors (“invalid condition”). The post-cue always had the same color as the target.

In three separate sessions, participants completed 12 neutral cue blocks of 54 trials for a total of 648 neutral trials and 12 color cue blocks of 112 trials for a total of 1344 color cued trials. This yielded 672 trials for the valid condition and 84 trials per test color for the invalid condition.

### Analysis: Boundary estimation and test color generation

We used frequency of clicks (see Fig. 2b) on the color wheel to estimate individual participant’s color boundaries. For each of the six frequency distributions of a participant, we fitted a von Mises distribution and computed the mean as the boundary location^45^. Each individual’s own estimated boundaries were used to generate the set of test colors for the main task. The color of the attentional cue was set at the middle of the color category, defined as the midpoint between the two boundaries for that category. The test colors were sampled at 0°, ±15°, ±30°, ±45° and ±60° away from the color of the attentional cue on the color wheel.

### Analysis: Model fitting and comparison

The cueing effect was calculated as the difference in accuracy (measured as proportion of correct responses) between the color cue condition and neutral cue condition. We fitted both a monotonic model and a non-monotonic model to the average cueing effect using non-linear regression. The data range for model fitting was chosen such that it included one data point from the rebound just outside the suppressive surround. Because the suppressive surround was found at different cue-target offsets in different experiments, the fit range also varied accordingly. The monotonic model was implemented as a Gaussian function, which had three free parameters:

$$Pc=\frac{A}{w}{e}^{-\frac{{x}^{2}}{2{w}^{2}}}+b,$$

where *Pc* is the cueing effect, *x* is the cue-target offset, *w, A, and b* are the free parameters controlling the shape of the function. The non-monotonic model was implemented as a negative second derivative of a Gaussian function, which has a Mexican hat shape:

$$Pc=\frac{2A}{\sqrt{3w}{\pi }^{\frac{1}{4}}}{e}^{-\frac{{x}^{2}}{2{w}^{2}}}(1-\frac{{x}^{2}}{{w}^{2}}),$$

where *Pc* is the cueing effect, *x* is the cue-target offset, *w* and *A* are the two free parameters controlling the model’s shape. Both Bayesian information criterion^46^ (BIC) and Akaike information criterion^47^ (AIC) were computed as evidence supporting each model. BIC was computed with the assumption of a normal error distribution:

$$BIC=n\,\mathrm{ln}(\frac{RSS}{n})+k\,\mathrm{ln}(n),$$

where *n* is the number of observations, *k* is the number of free parameters, and *RSS* is residual sum of squares^25^. We calculated the Bayes factor (*BF*) of the Mexican hat model over the Gaussian model based on BIC approximation^48^:

$$BF={e}^{(\frac{(BI{C}_{G}-BI{C}_{M})}{2})},$$

where *BIC*_*G*_ is for the Gaussian model, *BIC*_*M*_ is for the Mexican hat model. AIC was also calculated with the assumption of a normal error distribution^49^:

$$AIC=n\,\mathrm{ln}(\frac{RSS}{n})+2k,$$

Where *n* is the number of observations, *k* is the number of free parameters, and *RSS* is residual sum of squares. To compare the models, we calculated the likelihood ratio of the Mexican hat model over the Gaussian model^49^:

$$L={e}^{(\frac{(AI{C}_{G}-AI{C}_{M})}{2})},$$

where *AIC*_*G*_ is for the Gaussian model, *AIC*_*M*_ is for the Mexican hat model.

## Supplementary information

Supplementary_Materials_SR

## Data Availability

The data that support the finding of this study are available from the corresponding author upon request.
