# Supplementary material for: Attention to colors induces surround suppression at category boundaries
Source: Sci Rep. 2019 Feb 5;9:1443. doi: 10.1038/s41598-018-37610-7 (PMC6363742; doi:10.1038/s41598-018-37610-7)
Supplement: Supplementary file 1 — Supplementary_Materials_SR [file 41598_2018_37610_MOESM1_ESM.pdf]

Supplementary Materials

Attention to colors induces surround suppression at category boundaries

Ming W.H. Fang, Mark W. Becker, Taosheng Liu*

Neural model for surround suppression at categorical boundaries

Our goal here is to build the simplest model that is informed by physiological data to produce the attentional profile measured in our psychophysical experiments. The advantage of this approach, compared to a full-blown model such as multi-layered neural network model, is that we have a much better understanding of how model parameters impacts its behavior. However, simplicity is only achievable by ignoring many physiological details and as such, our model is more of a proof-of-concept than a complete description of the physiological processes. Nevertheless, such a model can still give useful insights regarding the neural mechanisms of attention.

The model contains a bank of identical, uniformly distributed, color-tuned neurons spanning the 360° space defined by the color wheel. Each neuron's tuning curve is assumed to be a circular Gaussian function (von Mises function)

$$R_{ij} = \frac{e^{\kappa \cos(\theta_j - \mu_i)}}{2\pi \, I_0(\kappa)} \cdot (A - s) \; + s \qquad (1),$$

where R_{ij} is the i-th neuron's response to a colored dot θ_j, κ is the concentration parameter that controls the spread of the tuning function, and μ_i is the neuron's preferred color. $I_0(\kappa)$ is the Bessel function of order 0. Parameter A denotes the firing rate to the preferred color, and s represents the spontaneous firing rate. The model contains 90 neurons with κ =12, s =10 spikes/s, A=40 spikes/s. The parameter values are based on relevant physiological findings (see Table S1 for the full list of parameters and their values). For simplicity, we assumed no neural noise or inter-neuronal correlation.

In a simulated trial of the 2-IFC task, the model is "shown" two random dot color stimuli, a noise pattern with random colors and a signal pattern at a particular color coherence. Each dot

independently evokes responses across all neurons, which are computed using Eq. (1). Each neuron's response is determined by averaging its responses to all dots in the stimulus, which is computed by

$$R_i = \frac{1}{N}\sum_{j=1}^{N} R_{ij} \qquad (2),$$

where N is the total number of color dots in each stimuli array (fixed at 100), and R_i is the neuron's average response to all dots. The response across all neurons to a dot array thus constitutes a population neural response and is the basis of the model's decision. For each stimulus interval, we fitted a Gaussian template (Eq. (1)) to the population response using maximum likelihood estimation (MLE), which had four free parameters – amplitude (A), mean (μ_i), variance (κ), and intercept (s). As the 2-IFC task requires participants to detect a stronger color signal, we used the estimated amplitude (A) as the decision criterion. The model simply chose the stimulus interval with a higher amplitude estimate as the target.

We first performed baseline simulations by presenting the model with color stimuli of different coherence levels. The model's choice for each trial was recorded and the proportion correct rate was calculated. For all results presented here, we simulated 2000 trials for each condition. In this baseline (neutral) condition, the model performed better with higher color coherence, similar to human observers (Fig. S1a). We also checked the population response for stimuli of different coherence levels and found it to increase monotonically with coherence (Fig. S1b). This increase in population response thus reflected the increase in the signal strength and was appropriately registered by the model. For the main simulations, we fixed the color coherence at 0.1 as it produces an intermediate performance level in the neutral condition. Indeed, this coherence level was comparable to coherence thresholds measured in our human participants (cf. Fig 5).

For attention condition, we first simulated the experiment with a pure feature-similarity gain modulation, which was implemented as a linear function:

$$\mathrm{G_{FSG}} = b - a * |(\theta_{attend} - \theta_{target})| \qquad (3),$$

where G_{FSG} is the gain factor, *b* denotes the intercept of the attentional gain, and *a* represents the slope. This equation expresses the FSG principle: the attentional gain factor for a target feature (i.e., θ_{target}) depends on its difference (similarity) to the attended feature (i.e., θ_{attend}). Without losing generality, we assumed θ_{attend} =0°. Values of *a* and *b* were based on published values from monkey MT (Martinez-Trujillo & Treue, 2004, see Table S1). The FSG modulation led to a simple scaling of all the tuning curves (Fig. S2a). To facilitate the understanding of the model behavior, we plotted the model's population response to a color signal under the attention and neutral condition for a few selected cue-target offset (Fig. S2b). As can be seen, compared to the neutral condition, population response for the 0° target was higher and gradually declined as target deviated more from 0° such that at large offsets, it became lower than the neutral condition. This monotonic decline of population response underlies the model's monotonic cueing effect (Fig. 8d).

Next, we implemented a hybrid model by combining the FSG modulation with neuronal tuning shifts (Fig. S3a). The FSG factor is calculated using Eq. 3 above. The magnitude of neurons' tuning shift towards the cued color, *M*, is calculated by a piece-wise linear function,

$$M = \begin{cases} 0.5 \cdot (\theta_i - \theta_{attend}), & if\ |\theta_i| \leq w, \\ 3 \cdot w \cdot sgn\ (\theta_i) - 2.5 \cdot \theta_i, & if\ w < |\theta_i| \leq 1.2w, \\ 0, & if\ 1.2w < |\theta_i| \leq 180° \end{cases} \tag{4}$$

where *w* denotes the boundary (40° in current case), θ_i is the neuron's original tuning preference, and θ_{attend} denotes the attended color (fixed at 0°), and *sgn* is the sign function. This results in a larger shift as neurons move further away from the attended feature followed by a reduced shift beyond the category boundary (see Fig. 8f). Once *M* declines to 0, the tuning shift would stop. Under this scenario, neuronal responses were calculated in the same fashion as in Eq. (1), except that neuron's preferred color (μ_i), was replaced by (μ_i-*M*), representing a shift in tuning preference.

The population responses under this hybrid modulation exhibited a non-monotonic profile. Critically, there was a suppression of population response for the boundary color compared to neutral baseline (Fig. S3b, 40°), which was not seen in the FSG only condition (cf. Fig. S2b,

40°). This was followed by a relative increase in population response at 60°, signifying a rebound. Finally, for large feature offsets such as 140°, there was a further suppression, as a result of FSG modulation. These qualitative observations on the population responses were registered by our model using the simple read-out rule described earlier, resulting in a hybrid of surround suppression and feature-similarity gain modulation in its performance (see Fig. 8g).

To verify whether surround suppression can appear at the categorical boundaries, we simulated the experiment with a number of different category width (e.g., ±30°, ±50° ±60°, ±70°, ±80° boundaries) and observed the suppressive surround occurring at the category boundaries. We also explored different shifting parameters that control the exact shape of the shifting function (Fig. 8f) and found that as long as the tuning shift returns to zero beyond the category boundary relative quickly, the model produces a surround suppression at the boundaries. For example, the slope of the declining portion of tuning shift beyond the boundary can be shallower. We also used a sinusoidal tuning shifting function and found similar results with the piece-wise linear function in Eq. 4.

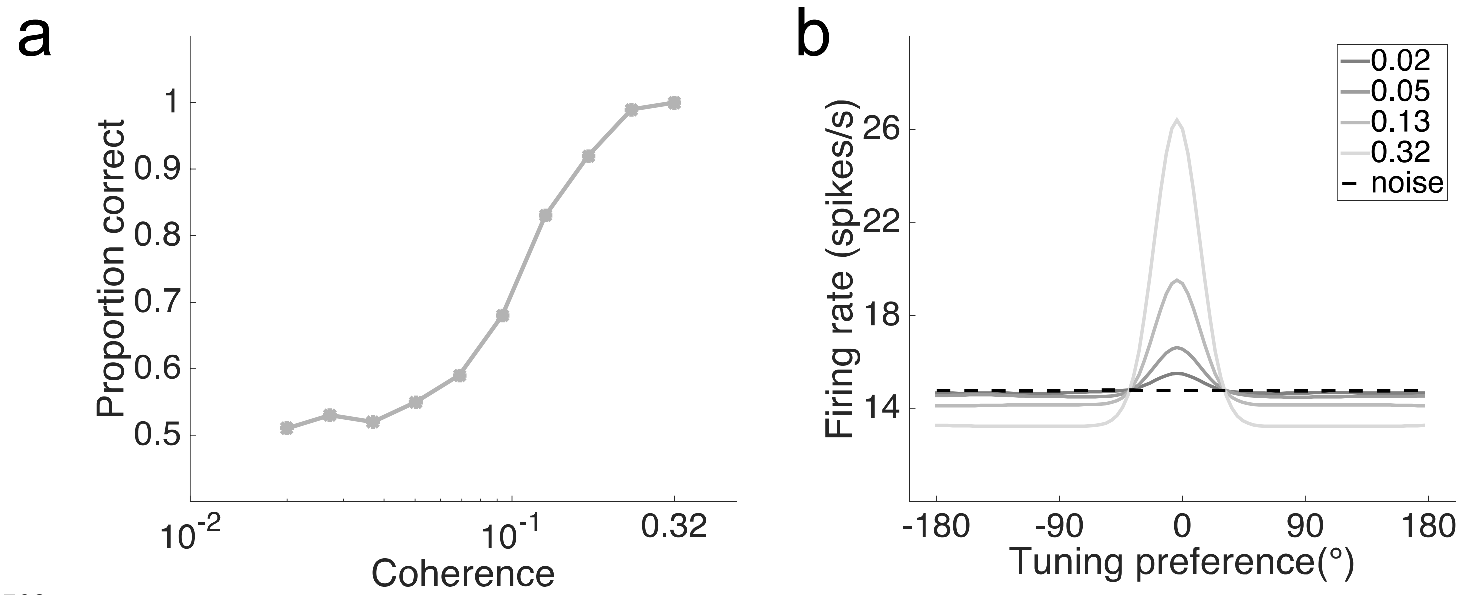

Figure S1. The model's neutral (baseline) performance and population responses under variable coherence levels. a). The model performed better as the coherence of color stimuli increased. b) Average population response across trials for a few selected coherence levels (gray lines). The dashed black line denotes the average population response for the noise stimuli.

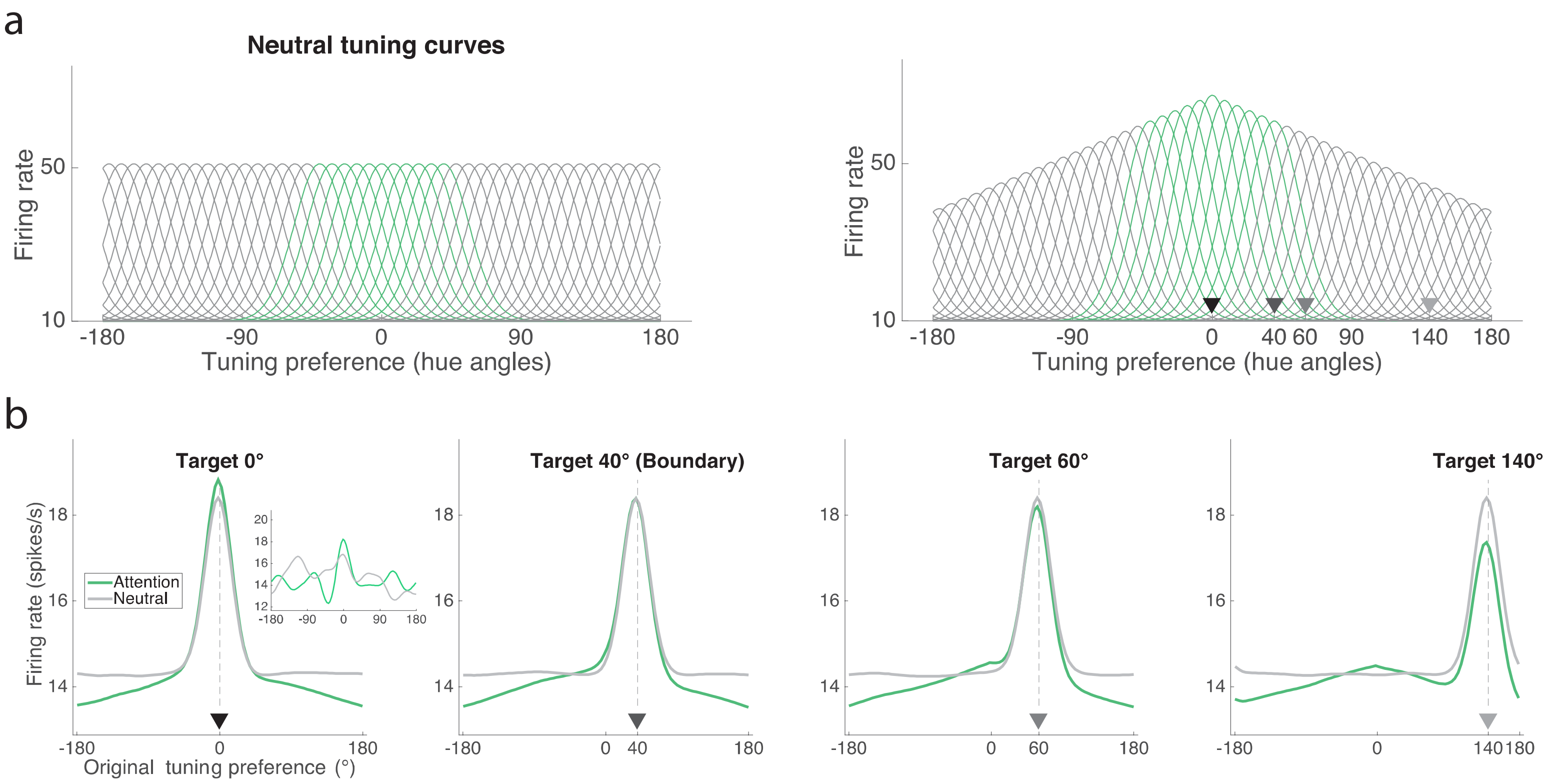

Figure S2. Pure FSG modulation on neural responses. **a)**. Groups of neuronal tuning curves in the neutral condition (left panel) and the attention condition due to FSG modulation (right panel). Attended feature is assumed to be at 0°. **b)**. Example average population responses to a color signal at four cue-target offsets. There was a monotonic decrease in the population responses (green curves) compared to neutral baselines (gray curves). The population responses were averaged across 2000 trials and thus appear quite smooth. Population responses on individual trials were much noisier. The inset in the left most panel shows population responses on a single trial.

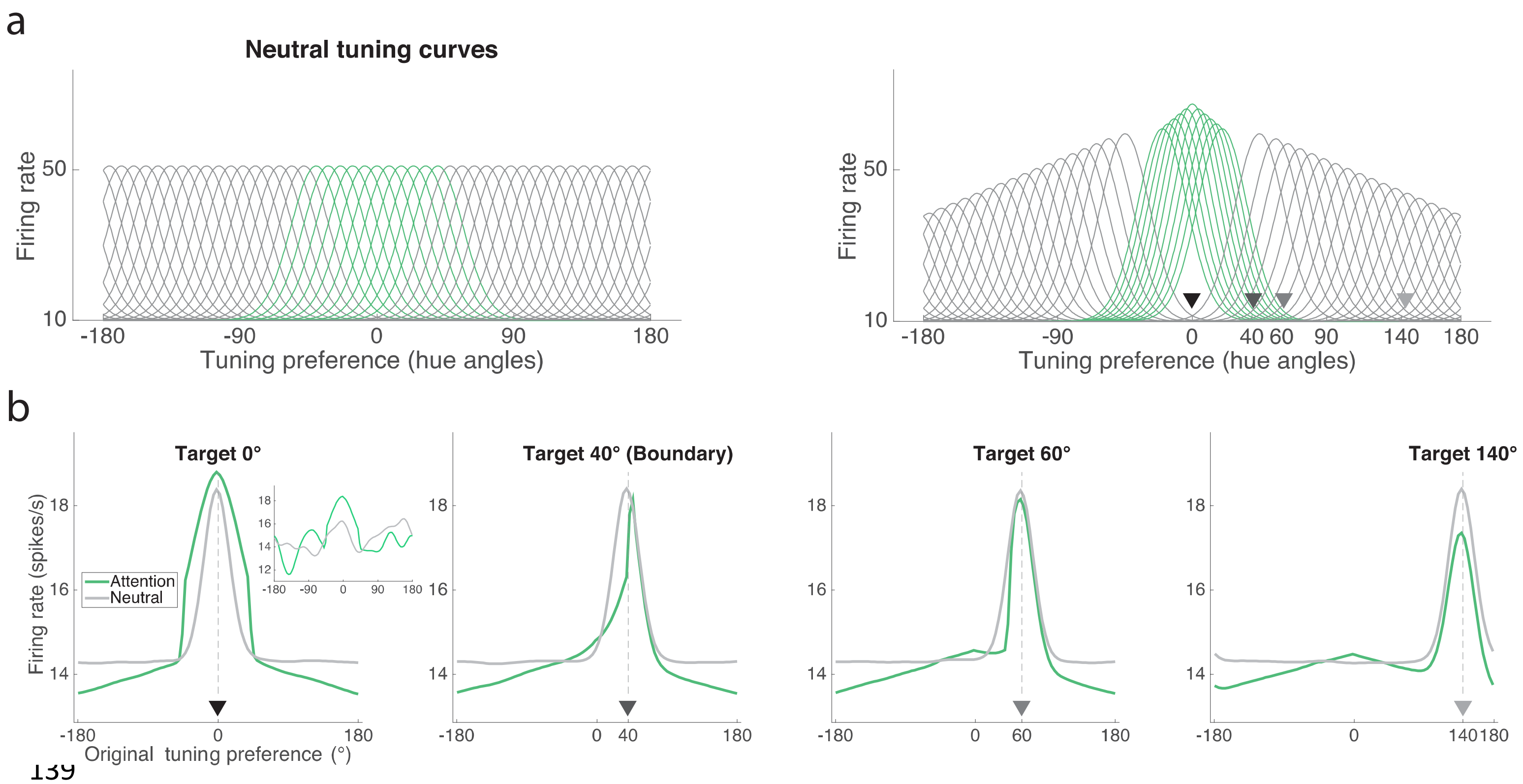

Figure S3. Combined modulation of FSG and tuning shift on neural responses. **a)**. In addition to FSG modulation, neurons also shifted toward the category center. **b)**. Example population responses to a color signal at four cue-target offset. Note the population responses in the attention condition (green curves) changed non-monotonically compared to neutral baselines (gray curves). The smooth population responses were averaged across 2000 trials. Examples of population responses on a single trial are shown in the inset of the left most panel.

Table 1. Parameters and their values used in the neural model simulation.

Name	Value	Description
κ	12	Single neuron's tuning bandwidth, equivalent to a bandwidth of ~39°, similar to previously reported value (Conway, Moeller, & Tsao, 2007)
b	1.0372	Intercept of attentional gain factor in the FSG model, based on values reported by Martinez-Trujillo, & Treue (2004)
a	0.00093	Slope of attentional gain factor in the FSG model, based on values reported by Martinez-Trujillo, & Treue (2004)
N	100	Number of colored dots in the simulation
s	10 spikes/s	Neuron's spontaneous firing rate
A	40 spikes/s	Neuron's firing rate to its preferred color
